# The Combination of Bioinformatics Analysis and Untargeted Metabolomics Reveals Potential Biomarkers and Key Metabolic Pathways in Asthma

**DOI:** 10.3390/metabo13010025

**Published:** 2022-12-23

**Authors:** Fangfang Huang, Jinjin Yu, Tianwen Lai, Lianxiang Luo, Weizhen Zhang

**Affiliations:** 1 Department of Respiratory and Critical Care Medicine, Nanfang Hospital, Southern Medical University, Guangzhou 510515, China; 2 Graduate School, Guangdong Medical University, Zhanjiang 524023, China; 3 The First Clinical College, Guangdong Medical University, Zhanjiang 524023, China; 4 Department of Respiratory and Critical Care Medicine, Affiliated Hospital of Guangdong Medical University, Zhanjiang 524001, China; 5 The Marine Biomedical Research Institute of Guangdong Zhanjiang, Guangdong Medical University, Zhanjiang 524023, China

**Keywords:** asthma, untargeted metabolomics, bioinformatics analysis, HIF-1a

## Abstract

Asthma is a complex chronic airway inflammatory disease that seriously impacts patients’ quality of life. As a novel approach to exploring the pathogenesis of diseases, metabolomics provides the potential to identify biomarkers of asthma host susceptibility and elucidate biological pathways. The aim of this study was to screen potential biomarkers and biological pathways so as to provide possible pharmacological therapeutic targets for asthma. In the present study, we merged the differentially expressed genes (DEGs) of asthma in the GEO database with the metabolic genes obtained by Genecard for bioinformatics analysis and successfully screened out the metabolism-related hub genes (HIF1A, OCRL, NNMT, and PER1). Then, untargeted metabolic techniques were utilized to reveal HDM-induced metabolite alterations in 16HBE cells. A total of 45 significant differential metabolites and 5 differential metabolic pathways between the control group and HDM group were identified based on the OPLS-DA model. Finally, three key metabolic pathways, including glycerophospholipid metabolism, galactose metabolism, and alanine, aspartate, and glutamate metabolism, were screened through the integrated analysis of bioinformatics data and untargeted metabolomics data. Taken together, these findings provide valuable insights into the pathophysiology and targeted therapy of asthma and lay a foundation for further research.

## 1. Introduction

Asthma is a complex chronic airway inflammatory disease involving multiple cells and cellular components that affects more than 300 million people worldwide, with an expected increase to 400 million by 2025 [1,2]. The pathophysiology of asthma is characterized by airway inflammation, airway hyper-responsiveness, eosinophil infiltration, reversible airflow obstruction, airway remodeling, mucus hypersecretion, and goblet cell hyperplasia [3,4]. However, the pathogenesis of asthma is still not well understood and is considered to relate to multiple factors, including genetics, environment, infection, immunity and nutrition [5]. Although glucocorticoids are the most effective drugs to control airway inflammation in asthma, the long-term use of high-dose inhaled glucocorticoids cause systemic adverse reactions that affect the growth and development of children [6,7]. Therefore, it is very important to understand the potential molecular pathogenesis of asthma and explore new effective targeted therapies.

Metabolomics is a rapidly developing field of life sciences which uses advanced analytical chemistry techniques and sophisticated statistical methods to comprehensively characterize small molecule (<1500 Da) metabolites found in specific cells, organs, or organisms [8,9]. Metabolites are the result of biological and environmental factors and have great potential to link knowledge of genotype and phenotype [10]. Recently, with the emergence and evolution of metabolomics technology, the discovery of the active metabolites with the ability to change cell physiology has increased rapidly [11]. In addition, Metabolomics is the omics field that is closest to phenotype expression and is a technique for monitoring the interaction between environment and host since measured metabolites reflect the changes of metabolic fluxes of various organs and cells [12,13]. Thus, metabolomics provides the potential for identifying biomarkers of asthma host susceptibility and elucidating biological pathways.

In this study, DEGs of asthma from the GEO database were intersected with metabolic genes obtained by Genecard for bioinformatics analysis and metabolism related DEGs were screened out. Then, we established an in vitro model of asthma and untargeted metabolic techniques were utilized to reveal HDM-induced metabolite alterations in 16HBE cells. Finally, through the integrated analysis of bioinformatics data and untargeted metabolomics data, this study identified the potential biomarkers and key metabolic pathways of asthma.

## 2. Materials and Methods

### 2.1. Data Download

To investigate the relationship between asthma and metabolism, the Gene Expression Omnibus (GEO database, https://www.ncbi.nlm.nih.gov/GEO/ (accessed on 29 April 2022) [14] was used to collect two databases related to asthma. We used the GSE76262 (Supplementary Materials, Table S1) as a training set and the GSE4302 (Supplementary Materials, Table S2) as a testing set. The top 1000 highly expressed metabolic genes were collected from GeneCard (https://www.genecards.org/ (accessed on 29 April 2022) [15].

### 2.2. Identify Molecular Subtypes Using NMF Algorithm

GSE76262 was clustered using the Non-Negative Matrix Decomposition (NMF) clustering algorithm. The standard “brunet” option was selected and 50 iterations were conducted for the NMF method. The number of clusters k was set from 2 to 7, the average contour width of the common membership matrix was confirmed by the R package “NMF” (version: 0.23.0), and the minimum member of each subclass was set to 10. The optimal number of clusters was determined by the cophenetic, dispersion, and silhouette indicators, and the optimal number of clusters was chosen to be 3.

### 2.3. The screening of Differentially Expressed Genes (DEGs)

The GSE76262 matrix data were normalized, log2 transformed, and screened for DEGs using the R package “Limma” (Version: 3.48.3). |log2FC| > 0.5 and the adjusted *p* value < 0.05 was considered as the threshold level of statistical significance for the samples of DEGs.

### 2.4. Time Series Analysis and the Intersection of Metabolic Genes and DEGs

Genes from the 3 subgroups obtained from NMF were grouped into different clusters using the R package “Mfuzz” (version: 2.52.0) and the fuzzy c-means algorithm. The genes from different clusters obtained by time series analysis were intersected with the top 1000 metabolic genes and DEGs.

### 2.5. LASSO Regression and Correlation Analysis

Receiver operating characteristic curves were drawn and the diagnostic value of key genes was assessed by their AUCs, and the genes and samples screened by intersection were used to build a binomial LASSO model using the R package “glmnet” (version: 4.1.3). As λ increases, LASSO tends to reduce the regression coefficients to zero. The genes were screened according to the λ value of the model whose evaluation index was within 1 standard error of the optimal value, namely, lambda.1se. Furthermore, to better determine the relationship between genes, correlation analysis was performed using Pearson’s correlation and visualized by employing the R package “corrplot” (version: 0.91).

### 2.6. Random Forest

Random forest (RF) is an ensemble machine learning method based on a decision tree algorithm that can be used for classification and regression-based analysis. The RF model was constructed by the R package “randomForest” (Version: 4.7.1). About 75% of the GSE76262 samples were used for training, while the remaining 25% were used for model testing. Meanwhile, the dataset GSE4302 was used as a test set to verify the feasibility of the model. The Boruta dimensionality reduction method was also applied in order to automatically select the important features generated by the above ranked list RF.

### 2.7. ROC Analysis of Risk Models

Based on the expression level of the sample, the RiskScore of each sample was calculated and the RiskScore distribution of the sample was plotted. Furthermore ROC analysis of RiskScore prognosis classification was performed using the R package “pROC” (version: 1.18.0).

### 2.8. Gene Expression and Hub Gene Screening

The high and low expression levels of genes in the training set GSE76262 and the test set GSE4302 were visualized using the R package “ggplot2” (version: 3.3.5). At the same time, a DEG PPI network was constructed using the Interactive Gene Retrieval Online Search Tool (STRING, https://www.string-db.org/ (accessed on 6 May 2022) database with a PPI score threshold (medium confidence) ≥ 0.4. The Cytohub plugin in Cytoscape (version 3.8.1, https://cytoscape.org/ (accessed on 6 May 2022) was used to score genes using ten algorithms of EcCentricity, DMNC, BottleNeck, EPC, Closeness, MNC, Radiality, and Degree. The gene with the highest score was determined to be the most hub gene.

### 2.9. Functional Enrichment Analysis

Statistically significant DEGs were further analyzed using the R package “BioManager” (version: 1.30.16) and “GOplot” (version: 1.0.2) for Gene Ontology (GO) function, Kyoto Gene, Encyclopedia of Genomes (KEGG) pathway enrichment.

### 2.10. Identification of Infiltrating Immune Cells in Asthma Samples

The CIBERSORT (https://cibersortx.stanford.edu/ (accessed on 14 May 2022) deconvolution algorithm was used to assess differential immune cell infiltration between asthma and healthy samples. CIBERSORT is an analytical tool that uses gene expression data to estimate the abundance of member cell types in mixed cell populations. The LM22 gene file offered by CIBERSORT was used to define and infer relative proportions in the 22 infiltrating immune cell Asthma gene expression data. The algorithm uses a default signature matrix of 100 permutations. To ensure confidence in the results, CIRBERSORT uses Monte Carlo sampling to deduce the deconvolution *p* value for each sample, and only holds data with *p* values < 0.05. The results obtained with CIBERSORT were visualized using the R package “ggplot2” and “ggpubr” (vversion: 0.4.0).

### 2.11. Reagents and Antibodies

GAPDH antibody was obtained from Sangon Biotech (Shanghai, China). E-cadherin and β-catenin antibodies were purchased from abcam (Shanghai, China). HIF-1a antibody was obtained from ABclonal (Wuhan, China). Alexa Fluor 488-conjugated secondary antibody was purchased from Thermo Fisher Scientific (Shanghai, China). House dust mite (HDM) was purchased from Greer (Beijing, China). Ammonium acetate (NH4AC) was purchased from Sigma Aldrich (St. Louis, MO, USA). Acetonitrile was purchased from Merck (Shanghai, China), and ammonium hydroxide (NH4OH) and methanol were purchased from Fisher.

### 2.12. Cell Culture and Treatment

Human bronchial epithelial cells (16HBE) were purchased from the Type Culture Collection of the Chinese Academy of Sciences (Shanghai, China). 16HBE cells were cultured in MEM medium (Gibco, Grand Island, NY, USA) containing 10% fetal bovine serum (FBS; Gibco, Grand Island, NY, USA) and 1% penicillin–streptomycin (Gibco, Grand Island, NY, USA) and incubated at 37 °C and 5% CO_2_. 16HBE cells were seeded at a density of 2 × 10^5^ cells/well in 6-well plates. On the following day, the cells were treated with serum-free MEM medium for 12 h, and then divided into a control group and an HDM group. Cells were treated with HDM (50 μg/mL) for 24 h in the HDM group, and no treatment was given to the control group.

### 2.13. Western Blotting

Proteins were extracted from cells and quantified using BCA protein assay kit (Sangon Biotech, Shanghai, China). Proteins were separated by 10% SDS-PAGE gels and transferred to nitrocellulose membranes. The membranes were blocked with 5% bovine serum albumin (BSA) for 1 h before incubating with primary antibodies at 4 °C overnight. After 13–15 h, the membrane was washed with TBST 3 times and incubated with horseradish peroxidase-labeled secondary antibody (1:4000) at room temperature for 1 h. Finally, BeyoECL Moon Kit (Beyotime Biotechnology, Shanghai, China) was added for color development and image J software was used to analyze the absorbance value of the images.

### 2.14. Immunofluorescence

Cell culture supernatants were removed and then 16HBE cells were washed twice with PBS. Fixation was performed with 4% paraformaldehyde at room temperature for 20 min and then permeabilized with 0.5% Triton-X100 for 15 min. Then, cells were incubated overnight at 4 °C with anti-E-cadherin antibody or anti-β-catenin antibody. After 20–24 h, cells were incubated with Alexa Fluor 488-conjugated secondary antibody at room temperature for 1 h, and DNA was stained with 4,6-diamidino-2-phenylindole (DAPI).

### 2.15. Untargeted Metabolomics Sample Collection and Preparation

16HBE cells were seeded at a density of 7 × 10^5^ cells/well in 10 cm^2^ plates. On the following day, the cells were treated with serum-free MEM medium for 12 h and then divided into a control group and an HDM group. Cells were treated with HDM (50 μg/mL) for 24 h in the HDM group (*n* = 4) and no treatment was given to the control group (*n* = 4). The culture medium from the cultured 16HBE cells was removed using a pipette. Then, the cells were washed with PBS under 37 °C. An amount of 800 μL of cold methanol/acetonitrile (1:1, *v*/*v*) was used to remove the protein and extract the metabolites. The mixture was collected into a new centrifuge tube and centrifuged at 14,000× *g* for 5 min to collect the supernatant. A vacuum centrifuge was used to dry the supernatant. Then, samples were re-dissolved in 100 μL of acetonitrile/water (1:1, *v*/*v*) solvent for LC-MS analysis. In order to monitor the repeatability and stability of instrument analysis, quality control (QC) samples were prepared by combining 10 μL of each sample and analyzed together with the other samples. The QC samples were inserted regularly and analyzed every 5 samples.

### 2.16. LC-MS/MS Analysis

Analysis was performed using an UHPLC (1290 Infinity LC, Agilent Technologies, Santa Clara, CA, USA) coupled to a quadrupole time-of-flight (AB Sciex TripleTOF 6600) in Shanghai Applied Protein Technology Co., Ltd. (Shanghai, China).

For HILIC separation, a 2.1 mm × 100 mm ACQUIY UPLC BEH 1.7 µm column (Waters, Dublin, Ireland) was used to analyze the samples. In both ESI positive and negative modes, the mobile phase contained A = 25 mM ammonium acetate and 25 mM ammonium hydroxide in water and B = acetonitrile. The gradient was 85% B for 1 min and was linearly reduced to 65% in 11 min; then, it was reduced to 40% in 0.1 min and kept for 4 min, and then increased to 85% in 0.1 min, with a 5 min re-equilibration period employed.

The samples were analyzed using a 2.1 mm × 100 mm ACQUIY UPLC HSS T3 1.8 µm column (Waters, Dublin, Ireland) for RPLC separation. In ESI positive mode, the mobile phase contained A = water with 0.1% formic acid and B = acetonitrile with 0.1% formic acid. In ESI negative mode, the mobile phase contained A = 0.5 mM ammonium fluoride in water and B = acetonitrile. The gradient was 1%B for 1.5 min and was linearly increased to 99% in 11.5 min and kept for 3.5 min. Then, it was reduced to 1% in 0.1 min and a 3.4 min re-equilibration period was employed. The gradients were at a flow rate of 0.3 mL/min, and the column temperatures were kept constant at 25 °C. An amount of 2 µL of each sample was used for injection.

The conditions of the ESI source were set as follows: Ion Source Gas1 (Gas1) as 60, Ion Source Gas2 (Gas2) as 60, curtain gas (CUR) as 30, source temperature as 600 °C, and IonSpray Voltage Floating (ISVF) as ±5500 V. In the MS only acquisition, the instrument was set to acquire over the *m*/*z* range 60–1000 Da, and the accumulation time for the TOF MS scan was set at 0.20 s/spectra. In auto MS/MS acquisition, the instrument was set to acquire over the *m*/*z* range 25–1000 Da, and the accumulation time for product ion scan was set at 0.05 s/spectra. The product ion scan was performed using information-dependent acquisition (IDA) in the selected high-sensitivity mode. The parameters were set as follows: the collision energy (CE) was fixed at 35 V with ± 15 eV; declustering potential (DP), 60 V (+), and −60 V (−); exclude isotopes within 4 Da; and candidate ions to monitor per cycle: 10.

### 2.17. Untargeted Metabolomics Data Processing

The raw MS data (wiff.scan files) were imported into XCMS software after being converted to MzXML files using ProteoWizard MSConvert. The following parameters were used for peak picking: centWave *m*/*z* = 25 ppm, peakwidth = c (10, 60), and prefilter = c (10, 100). For peak grouping, bw = 5, mzwid = 0.025, and minfrac = 0.5 were used. CAMERA (Collection of Algorithms of MEtabolite pRofile Annotation) was sued for annotation of isotopes and adducts. In the extracted ion features, only the variables having more than 50% of the nonzero measurement values in at least one group were kept. Compound identification of metabolites was performed by comparing accuracy *m*/*z* value (<25 ppm) and MS/MS spectra with an in-house database established with available authentic standards.

### 2.18. Combined Analysis of Bioinformatics Analysis and Untargeted Metabolomics

The common pathways from network pharmacology and metabolomics were selected by Venn diagram.

### 2.19. Statistical Analysis

Data are presented as mean ± standard deviation (SD). Statistical analysis was performed using GraphPad Prism 8.0.1 analysis software. Differences between two groups were assessed using t test. A value of *p* < 0.05 indicates statistically significant difference; * indicates *p* < 0.05. For the statistical analysis of untargeted metabolomics results, the data were analyzed by R package (ropls) after sum-normalization, where it was subjected to multivariate data analysis, including Pareto-scaled principal component analysis (PCA) and orthogonal partial least squares discriminant analysis (OPLS-DA). The robustness of the model was evaluated by 7-fold cross-validation and response permutation testing. The variable importance in the projection (VIP) value of each variable in the OPLS-DA model was calculated to indicate its contribution to the classification. The significance of differences between the two groups of independent samples was determined by Student’s t test. The criteria for screening significant changed metabolites were VIP > 1 and *p* value < 0.05. The correlation between two variables was determined by Pearson’s correlation analysis.

## 3. Results

### 3.1. Molecular Typing and Identification of DEGs

**Figure 1:**
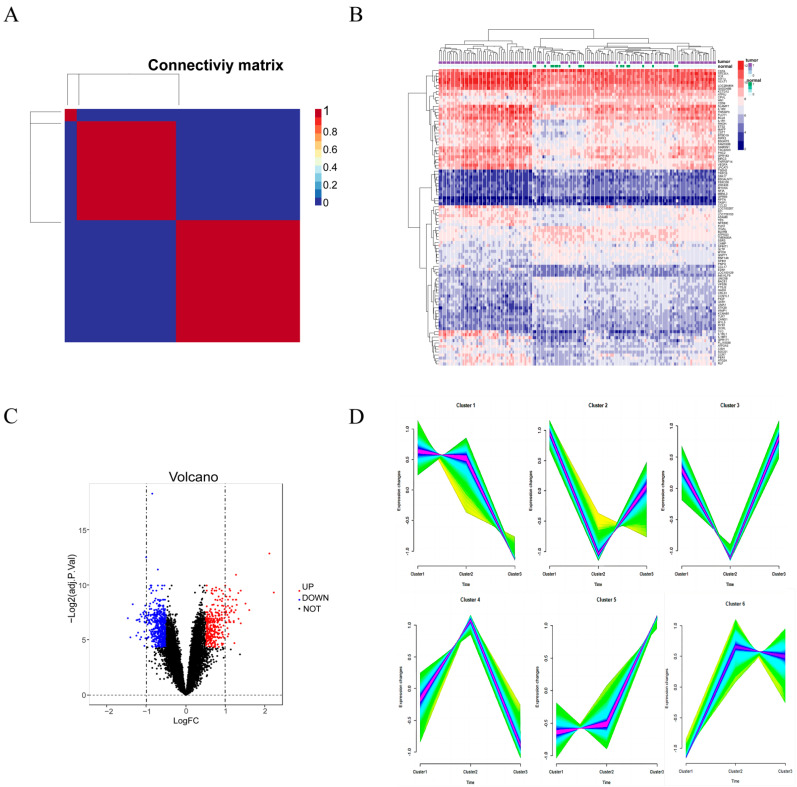
Differential expressed genes analysis. (**A**) Consensus map of NMF clustering. (**B**) The heatmap shows the expression of the 100 most differentiated genes in individuals. Green is the normal group, purple is the tumor group; the blue square represents low expression and the red square represents high expression. (**C**) The volcano plot shows upregulated and downregulated genes. (**D**) Fuzzy c-means clustering identified time series patterns of expression for the three clusters. The *x*-axis represents the three clusters, while the *y*-axis represents the normalized intensity ratio of log2 turns for each stage.

GSE76262 was selected and the NMF algorithm was used to identify molecular subtypes. According to the cophenetic, dispersion, and silhouette indicators, the optimal clustering number of 3 was selected (Figure 1A and Figure S1). As shown in the Supplementary Materials, Figure S2A, it can be seen that different NMF classes have their own weighted signatures. The R package “Limma” was used to further perform DEGs analysis on GES76262. A total of 928 DEGs (Supplementary Materials, Table S3) in the GSE76262 were screened, including 333 upregulated and 595 downregulated genes (|log2FC| > 0.5 and adjusted *p* value < 0.05) (Figure 1C). As can be seen in Figure 1B, the expression levels of upregulated and downregulated genes are presented in the heatmap, and these genes clustered well across the normal and diseased groups.

### 3.2. Time Series Analysis and Take Intersection

**Figure 2:**
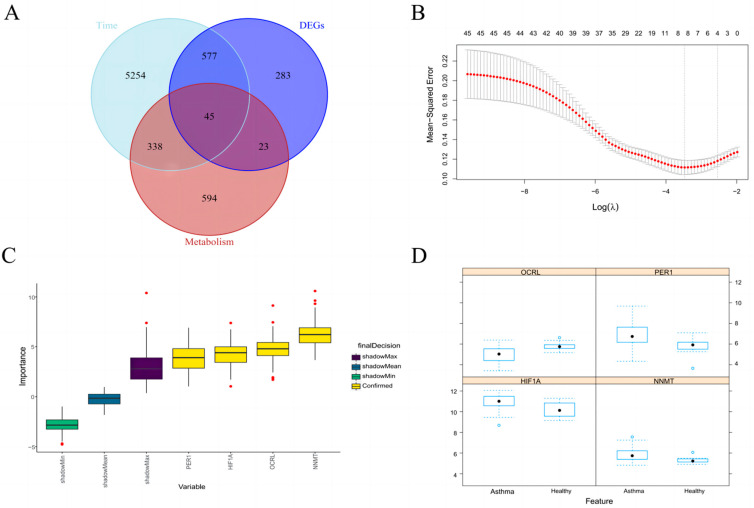
Intersected genes and further screening of genes. (**A**) Venn figure for intersected genes in time series analysis, metabolic genes, and DEGs. (**B**) The gene signature selection of optimal parameter (lambda) in LASSO model; the dashed line on the right indicates the λ value of the model with the evaluation index in the best value of 1 standard error range, lambda.1se. (**C**) The expression of module genes in the dataset GSE76262. (**D**) Importance of each factor in the RF model based on set GSE76262.

We applied the fuzzy c-means algorithm to cluster the three subtypes obtained from NMF typing. As shown in Figure 1D, we observed six distinct clusters of subtype patterns, representing differently regulated genes, indicating distinct expression dynamics. Among them, cluster 1 represents downregulated genes, cluster 5 represents upregulated genes, and clusters 2, 3, 4, and 6 represent genes showing a bimodal expression pattern. Therefore, we selected the genes of cluster 1 and cluster 5 to further intersect with the top 1000 metabolic genes collected on Genecard and 928 DEGs, as shown in Figure 2A, to obtain a total of 45 upregulated or downregulated genes related to metabolism.

### 3.3. Identification of Key Metabolic Gene Associated with Asthma

We performed LASSO regression analysis on the 45 genes as shown in Figure 2B. Using 1-s.e. as the standard, we identified four genes as HIF1A, NNMT, OCRL, and PER1. We also analyzed the correlation between these four genes (|log2FC| > 0.5 and adjusted *p* value < 0.05). A bitmap of the correlation analysis between genes is shown in the Supplementary Materials, Figure S2B. Blue and red indicate positive and negative correlations, respectively. The darker the color, the higher the correlation coefficient. There was a significant positive correlation among HIF1A, NNMT, and PER1, and they were highly negatively correlated with OCRL.

### 3.4. Random Forest further Screen Hub Genes

**Figure 3:**
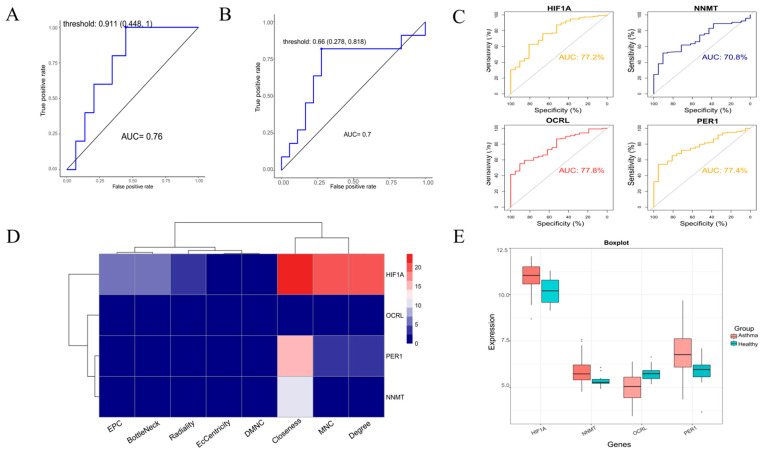
Verify model feasibility and visualize gene expression. (**A**) Model evaluation based on training set GSE76262. (**B**) Model evaluation based on test set GSE4302. (**C**) ROC curves of HIF1A, NNMT, OCRL, and PER1 in datasets GSE76262. (**D**) The 8 scoring methods of PPI to score HIF1A, NNMT, OCRL, and PER1. (**E**) The expression of HIF1A, NNMT, OCRL, and PER1 in the training dataset GSE76262.

We subjected these four genes to a Boruta feature selection “random forest” analysis (*p* < 0.05), selected to identify key categorical variables, as shown in Figure 2C, with NNMT showing the most important and HIF1A ranking third. Important variables and potentially important variables were then extracted; as shown in Figure 2D, HIF1A, NNMT, OCRL, and PER1 were chosen. HIF1A scored higher than NNMT in the feature selection, indicating that it is an important variable. Finally, the final selection model was extracted and the ROC curve evaluation model of the training set GSE76262 and the test set GSE4302 were drawn. The overall AUC value of the model was calculated, as shown in Figure 3A,B, and the AUC values of GSE76262 and GSE4302 were 0.76 and 0.7, which means the model has high feasibility.

### 3.5. Model Validation and Expression Level Analysis of Hub Genes

We performed ROC analysis on the four genes of HIF1A, NNMT, OCRL, and PER1 as shown in Figure 3C. The ROC curve revealed the probability of these four genes as valuable biomarkers and the results showed that OCRL and the maximum AUC values of HIF1A were 77.8% and 77.2%, respectively. Then, the eight scoring methods of PPI analysis were used to score the four genes, as can be seen in Figure 3D. The results showed that the highest score of HIF1A was 23.5 points, while OCRL scored much lower than HIF1A. Therefore, HIF1A was determined to be the most hub gene, and as shown in Figure 3E and in the Supplementary Materials, Figure S2C, the expression level of HIF1A was significantly upregulated in the training set GSE76262 and the test set GSE4302 compared with other genes.

### 3.6. Function and Pathway Enrichment Analysis

**Figure 4:**
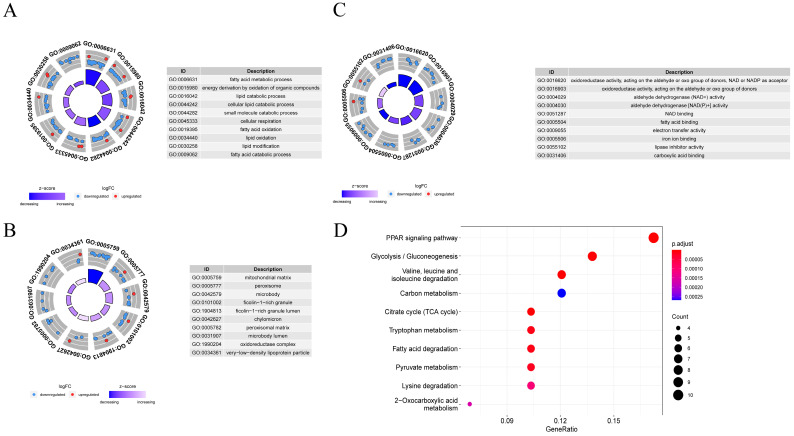
Functional enrichment analysis. (**A**–**C**) Gene Ontology (GO) enrichment analysis, including biological process (BP), cell composition (CC) and molecular function (MF). (**D**) Kyoto Encyclopedia of Genes and Genomes (KEGG) enrichment analysis.

GO analysis classified 68 genes related to metabolism into three categories: biological process (BP), molecular function (MF), and cellular component (CC). As shown in Figure 4A–C, BP-related genes are significantly enriched in fatty acid metabolic process, energy derivation by oxidation of organic compounds, and lipid catabolic process, which are involved in metabolic reactions. Genes linked to CC were significantly enriched in mitochondrial matrix, peroxisome, and microbody, which were closely related to cellular respiration. MF-related genes were significantly enriched in oxidoreductase activity, acting on the aldehyde or oxo group of donors, NAD or NADP as acceptor. As shown in Figure 4D, KEGG pathway enrichment analysis showed that genes were mainly enriched in the PPAR signaling pathway, glycolysis/gluconeogenesis, carbon metabolism, and citrate cycle (TCA cycle).

### 3.7. HIF1A-Enriched Pathways and Immune Infiltrates Analysis

In order to explore the specific pathway of HIF1A, we visualized the results, as shown in the Supplementary Materials, Figure S3A. The results show that in GO-BP, HIF1A is mainly enriched in energy derivation by oxidation of organic compounds and compounds cellular respiration. Then, we used CIBERSORT software to reveal patterns of immune cell infiltration with GSE76262. After data and processing screening, asthma samples and healthy samples in the GSE76262 dataset were included in the following analysis and a heat map was used to display the proportion of 22 immune cells in these 2 groups of samples. Macrophages.M0 and Mast.cells.activated represent the two most infiltrated fractions in the two groups. As shown in the Supplementary Materials, Figure S3B, the proportion of M0 macrophages in the asthma group was higher than the healthy group. Furthermore, the proportion of mast cells in the asthma group was lower than in the healthy group. This finding implies that metabolic-related asthma may have synergistic or antagonistic effects on immune cells such as macrophages.

### 3.8. HDM Induced Delocalization of E-cadherin and β-catenin and Promoted the Expression of HIF-1a in 16HBE Cells

**Figure 5:**
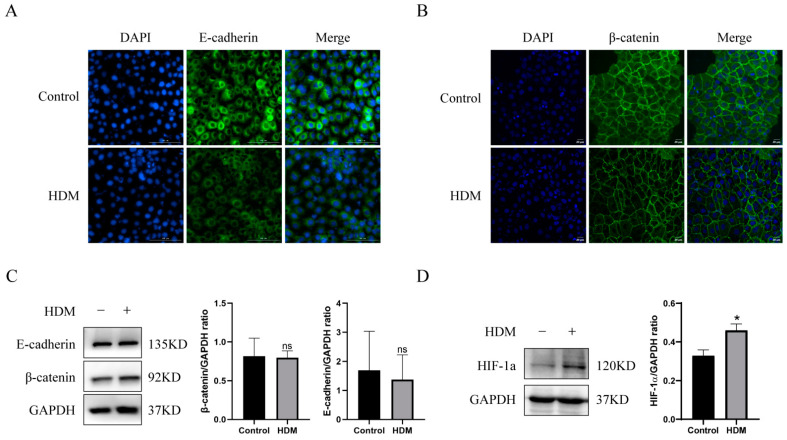
HDM induced delocalization of E-cadherin and β-catenin and promoted the expression of HIF-1a in 16HBE cells. (**A**,**B**) The distribution of E-cadherin and β-catenin were detected by immunofluorescence. (**C**,**D**) The expression of E-cadherin, β-catenin, and HIF-1a were detected by Western blotting. * Represents compared to the control group. * *p* < 0.05.

Next, the asthmatic model was established by challenging 16HBE (human bronchial epithelial) cell with house dust mite (HDM). First, we detected the effects of HDM on E-cadherin/β-catenin that mediate tight junctions in 16HBE cells using Western blot and immunofluorescence. Western blot results showed that HDM stimulation did not affect the expression of E-cadherin or β-catenin in 16HBE cells (Figure 5C). However, immunofluorescence results revealed that HDM promoted delocalization of E-cadherin and β-catenin in 16HBE cells, exhibiting discontinuous and diffusing from the cell membrane to the cytoplasm (Figure 5A,B). In addition, we investigated whether HDM affected HIF-1a expression in 16HBE cells. Western blotting revealed that HDM treatment increased the expression level of HIF-1a (Figure 5D). Therefore, these data indicated that the asthma model was successfully established and HDM induced delocalization of E-cadherin and β-catenin and promoted the expression of HIF-1a in 16HBE cells.

### 3.9. Multivariate Analysis of Metabolomic Data

**Figure 6:**
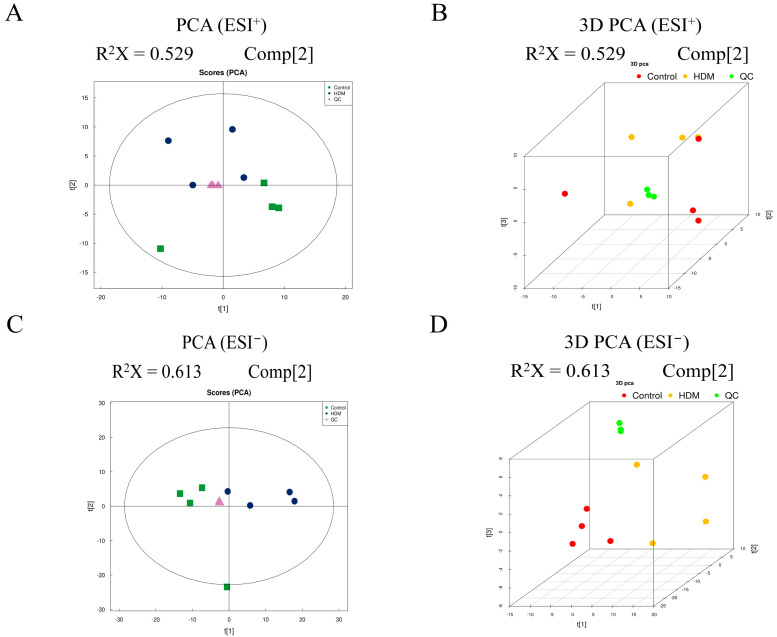
Principal component analysis (PCA) of metabolomic data. All samples in ESI+ modes are distributed in 2-dimensional plot (**A**) and in 3-dimensional plot (**B**). All samples in ESI− modes are distributed in 2-dimensional plot (**C**) and in 3-dimensional plot (**D**).

Raw data of LC-MS/MS in Wiff format were converted to mzXML format using ProteoWizard, and then peak alignment, retention time correction, and extracted peak areas were performed using XCMS software, followed by experimental data quality evaluation and multivariate statistical analysis. Principal component analysis (PCA) is an unsupervised data analysis method that can reflect the overall metabolic differences among each group and the variation degree among samples within the group. PCA analysis was performed for each comparison group, and the results showed that the data profile of the HDM group was far away from that of the control group, with significant separation between groups and high clustering within each group (Figure 6), which indicated that the metabolic network of the HDM group changed significantly. In addition, QC samples were closely clustered together in ESI positive (ESI+) and negative (ESI−) ion modes (Figure 6), indicating stability and repeatability of the analysis system.

**Figure 7:**
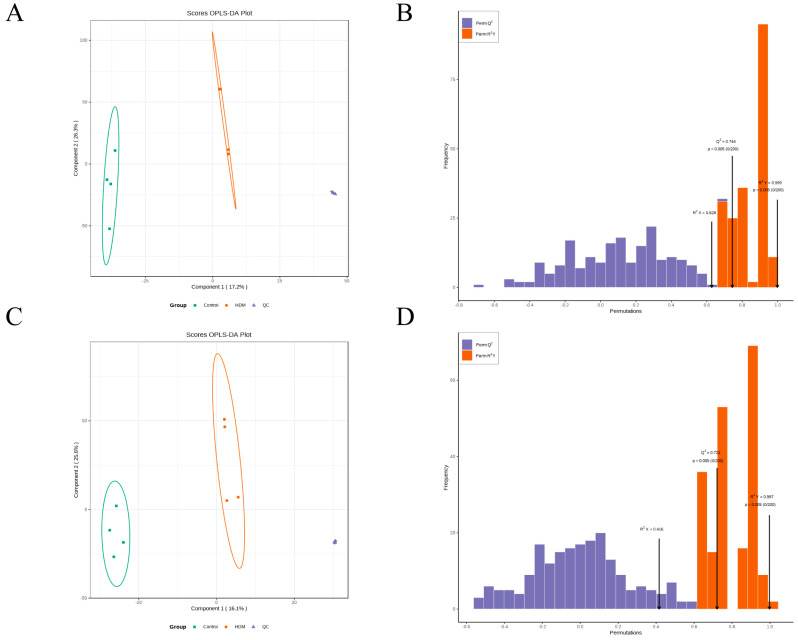
OPLS-DA score plots and permutation tests results. (**A**,**B**) OPLS-DA score plot and permutation tests result of metabolomics profiling in ESI+ mode. (**C**,**D**) OPLS-DA score plot and permutation tests result of metabolomics profiling in ESI− mode.

Orthogonal partial least squares discriminant analysis (OPLS-DA) is a supervised statistical method that can filter out noise unrelated to classification information and improve the analytical ability and effectiveness of the model. As shown in Figure 7A,C, the samples in the HDM group were clearly separated from those in the control group in OPLS-DA score plots either in ESI+ or ESI− modes, indicating that the HDM group has completely different metabolic characteristics compared with the control group. In this model, R2X and R2Y represented the interpretation rate of the X and Y matrices of the OPLS-DA model, respectively, and Q2 represented the predictive ability of the model. Among them, Q2 values reflected model predictability and exceeded 0.5 in either ESI+ or ESI− mode, indicating that good prediction ability and reliability of the model. In addition, permutation testing (*n* = 200) was performed to validate the validity of the supervised model. As shown in Figure 7B,D, the *x*-axis represented the accuracy of the model and the *y*-axis represented the frequency of the accuracy of the 200-permutation test. The *p* values of Q2 were less than 0.005, indicating that the prediction ability of the no randomization model in this permutation test were better than that of the OPLS-DA model. The *p* values of R2Y were less than 0.005, indicating that the interpretation rate of the Y matrix of the no randomization model in this permutation test were better than that of the OPLS-DA model. The abovementioned results verified the effectiveness of the model.

### 3.10. Identification of the Differential Metabolites

Variable importance for the projection (VIP) obtained by the OPLS-DA model can be used to measure the influence intensity and explanatory ability of the expression pattern of each metabolite on the classification and discrimination of each group of samples. Therefore, metabolites with VIP > 1 are considered to contribute significantly in model interpretation. In this study, we screened significant differential metabolites between the control group and the HDM group following the criteria of a *p* < 0.05 for the t-test and the VIP > 1.5 for the OPLS-DA model, and a total of 45 significant differential metabolites were screened (Table 1), including 12 metabolites in ESI+ mode and 33 metabolites in the ESI− mode. Compared with the control group, the levels of metabolites such as lactose and d-mannose increased, while the levels of metabolites such as carnitine, d-glutamine, glutamic acid, glycerophosphocholine, and phosphorylcholine decreased in the HDM group.

### 3.11. Metabolic Pathway Analysis

**Figure 8:**
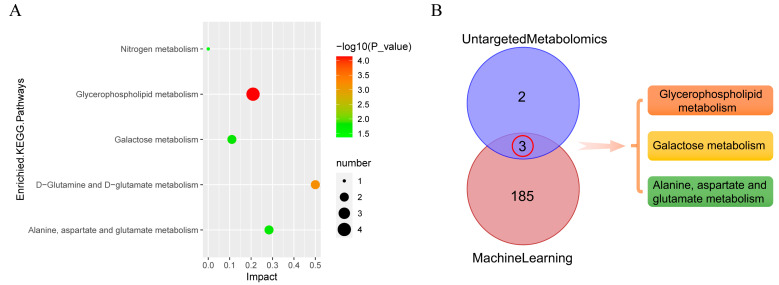
Metabolic pathway analysis. (**A**) The KEGG pathway bubble plot of significant differential metabolites. (**B**) The pathways co-enriched by bioinformatics analysis and metabolomics analysis.

Next, significant differential metabolites were further analyzed to investigate the effects of HDM stimulation on metabolic pathways in 16HBE cells. KEGG pathway enrichment analysis was performed by Fisher‘s Exact Test to analyze and calculate the significance level of metabolite enrichment of each pathway, so as to identify the significantly affected metabolic and signal transduction pathways. The smaller the *p* value, the more significant the difference in this metabolic pathway. In this study, the KEGG enrichment analysis results were ranked according to *p* value and five pathways were obviously changed (*p* value < 0.05) in the HDM group compared with the control group (Figure 8A). The significant enriched pathways were glycerophospholipid metabolism, d-glutamine and d-glutamate metabolism, galactose metabolism, alanine, aspartate and glutamate metabolism, and nitrogen metabolism. Then, to explore key metabolic pathways, 188 KEGG pathways enriched by bioinformatics analysis and 5 KEGG pathways enriched by untargeted metabolomics were intersected. As shown in Figure 8B, three common pathways, including glycerophospholipid metabolism, galactose metabolism, and alanine, aspartate, and glutamate metabolism, were obtained. In addition, significant differential metabolites (including d-glutamine, *N*-acetyl-l-aspartic acid, glutamic acid, glycerophosphocholine, phosphorylcholine, Myo-inositol, lactose, d-lactose, and melibiose) were enriched in three common pathways.

## 4. Discussion

Asthma is a chronic inflammatory disease characterized by airway hyper-responsiveness, airway inflammation, and airway remodeling [16]. At present, the pathogenesis of asthma remains not well understood and is considered to be associated with multiple factors such as genetics, environment, infection, immunity, and nutrition [5]. Therefore, there is an urgent need to identify biomarkers of pathophysiologic pathways to guide asthma treatment. In this study, comprehensive bioinformatics analysis was used to identify potential key genes associated with metabolism in asthma and untargeted metabolic techniques were also utilized to reveal HDM-induced metabolite alterations in 16HBE cells. Combined with the results of comprehensive bioinformatics analysis and untargeted metabolomics analysis, this study identified potential biomarkers and key metabolic pathways in asthma.

Unlike traditional biomedical research, machine learning is dedicated in learning natural patterns from massive amounts of high-throughput data and then using the natural patterns to predict unknown data, which is widely used in biological research [17]. Through machine learning and bioinformatics techniques, four hub genes, including HIF1A, NNMT, OCRL, and PER1, were identified as potential markers associated with asthma metabolism, especially HIF-1A. HIF-1α is a transcription factor that plays an important role in the regulation of functional inflammation and metabolic genes in mammalian cells. At present, many studies have reported that HIF-1α is also a key inflammatory regulator of asthma [18,19,20,21]. S. Huerta-Yepez, et al. demonstrated that HIF-1α is increased after challenges in patients with asthma and that HIF may be a potential therapeutic target for asthma [19]. In addition, it has been found that PM2.5 promoted the differentiation of TH17 cells by upregulating the expression of HIF-1α and enhancing glycolysis; thus, aggravating asthma [22]. Moreover, treatment with HIF-1α antagonist YC-1 can decrease airway hyper-responsiveness, blood eosinophilia, and allergic inflammatory gene expression in asthmatic mice [20].

Metabolomics is an important tool for medical research and has been widely used to identify new biomarkers and understand the molecular mechanisms of diseases [23,24]. Asthma has been found to be associated with metabolic abnormalities [25]. In a study using pediatric plasma for untargeted metabolomics analysis, children with mild to moderate asthma were distinguished from children with severe asthma by metabolic pathways associated with oxidative stress [26], which is consistent with the observation of increased oxidative stress in severe asthma [27]. In addition, several targeted metabolomics studies of plasma levels of polyunsaturated fatty acids (PUFAs) in children have reported a potential association between PUFAs levels and the risk of asthma and allergic diseases and the possible benefit of PUFAs supplementation on asthma and allergic disease prevention [28,29,30].

Airway epithelium is a dynamic orchestrator of immune response in Type 2 high asthma and is the main cell forming the barrier against mechanical stress, oxidative stress, allergens, pollutants, and infectious agents [31]. There are notable changes in the airway epithelium in patients with asthma including tight junction destruction and cell exfoliation, which promote epithelial damage and increased permeability across the epithelium [32,33,34]. In addition, dysfunctional airway epithelium in asthmatics could allow easier access of external pathogens to airway submucosa, which further leads to chronic airway inflammation related to asthma pathogenesis [32,35,36]. In this study, untargeted metabolic analysis was utilized to reveal HDM-induced metabolite alterations in 16HBE cells and further identify potential biomarkers and key metabolic pathways in asthma.

Through untargeted metabolomics analysis, we screened 45 differential metabolites and 5 metabolic pathways. Then, three key pathways, including glycerophospholipid metabolism, galactose metabolism, and alanine, aspartate, and glutamate metabolism, were selected by combined analysis of bioinformatics data with untargeted metabolomics data. In addition, significant differential metabolites (including d-glutamine, *N*-acetyl-l-aspartic acid, glutamic acid, glycerophosphocholine, phosphorylcholine, Myo-inositol, lactose, d-lactose, and melibiose) were enriched in three common pathways. In cellular physiological processes, glycerolphospholipid are essential structural components of biological membranes, lipoproteins, and pulmonary surfactant, and form phospholipid bilayers [37]. The profile of glycerolphospholipid is dramatically altered in asthmatic plasma, and glycerolphospholipid metabolism is associated with endoplasmic reticulum stress and oxidative stress in asthma pathogenesis [38]. It has been found that phosphorylcholine is a component of epithelial cell barrier, and the decrease in its level may indicate the lack of protection of the alveoli and airway [39]. In addition, it is noteworthy that glycerophosphocholine is the major glycerophospholipid in eukaryotic cellular membranes that participate in a variety of indispensable metabolic and intracellular signaling processes [40].

Previous studies showed that redox disorder and oxidative stress are related to the pathogenesis of asthma and may be a common mechanism in the phenotype and endotype of asthma [41,42]. It has been found that amino acids contribute to various antioxidant and immunological activities associated with asthma pathogenesis, in particular glycine, glutamine, and glutamate may have potentially protective effects [43,44]. In this study, significant differential metabolites (including d-glutamine, *N*-acetyl-l-aspartic acid, and glutamic acid) were enriched in three key metabolic pathways. Glutamine is the most abundant amino acid in the human body that participates in various cell biological processes, including redox homeostasis [45], energy and nucleotide formation [46], and glucose metabolism [47]. One study has reported that plasma glutamine levels were significantly lower in patients with mild asthma than in healthy controls [48]. Another study showed that glutamine administration effectively suppressed key features of Th2-dependent asthma, including airway eosinophilia, mucus formation, airway type 2 cytokine production, and late airway hyper-responsiveness by inhibiting cytosolic phospholipase A(2) activity in the airway in a murine asthma model [49]. Furthermore, other significant differential metabolites, such as carnitine, were also obtained by untargeted metabolomics analysis. Carnitine is a small water-soluble molecule with important physiological roles, including the transport of fatty acids into the mitochondrial matrix for beta-oxidation [50]. Studies showed that serum carnitine levels decrease in children with moderate asthma and a guinea pig model of allergic asthma [51,52].

## 5. Conclusions

In this study, four hub genes including HIF1A, OCRL, NNMT, and PER1 were identified as potential markers associated with asthma metabolism by bioinformatics analysis. Then, a total of 45 significantly differential metabolites were screened by further metabolomics analysis. Through the integrated analysis of bioinformatics data and metabolomics data, we screened three key metabolic pathways, involving glycerophospholipid metabolism, galactose metabolism, and alanine, aspartate, and glutamate metabolism. Our findings suggest that HDM plays a role in disturbing glycerophospholipid, galactose, and amino acids metabolism in 16HBE cells, and these changes may contribute to the development of asthma. Although more experiments are needed to confirm the results of this study, our results provide valuable insights into the pathophysiology and targeted therapy of asthma and lay a foundation for further research.

## Figures and Tables

**Table 1:** Significant differential metabolites identified by untargeted metabolomics analysis.

Adduct	Name	VIP	Fold Change	*p*-Value	PPM
[M + H] +	Carnitine	2.200291616	0.338584131	0.000426347	2.541680077
[M + H] +	Glu-Gly-Arg	1.818933829	4.591986615	0.000977944	0.869071719
[M + H] +	Palmitoyl sphingomyelin	4.642574354	0.476560728	0.001748888	0.153548353
[M + Na] +	Lactose	3.773556063	3.281279376	0.00259977	1.619460097
[M + H] +	d-lactose	1.857280581	2.851001893	0.003333963	3.055191786
[M + H-H_2_O] +	Melibiose	1.593316801	2.778378355	0.004764936	1.882063955
[M + H-NH_3_] +	d-glutamine	2.036482003	0.587719538	0.006265209	2.365700892
[M + H] +	Glycerophosphocholine	4.450704967	0.645349102	0.011104682	1.146858383
[M + H] +	4-(2-hydroxyethyl)piperazine-1-ethanesulfonic acid	9.213222731	1.284113383	0.015014811	0.536893891
[M + H] +	Phosphorylcholine	4.109895791	0.425042372	0.016706732	1.511398798
[M + H] +	Pro-Trp	1.571671351	0.757490368	0.017565433	1.702405805
[M + H] +	1-hexadecyl-2-(9z-octadecenoyl)-sn-glycero-3-phosphocholine	3.02416122	0.570834294	0.032006053	1.19605325
[M − H] −	(2e,6e,10e)-13-[(2r)-6-hydroxy-2,8-dimethyl-3,4-dihydrochromen-2-yl]-2,6,10-trimethyltrideca-2,6,10-trienoic acid	1.548347947	6.564924847	4.61073 × 10^−7^	2.932669011
[M − H] −	Mitragynine	1.548364808	4.037763655	1.42082 × 10^−6^	2.99940101
[M − H-HF] −	5-heptenoic acid, 7-[(1r,2r,3s,5s)-2-[(1e,3s)-3-(2,3-dihydro-1h-inden-2-yl)-3-hydroxy-1-propen-1-yl]-3-fluoro-5-hydroxycyclopentyl]-, (5z)-	1.753276464	6.597303608	1.60723 × 10^−6^	1.231379272
[M − H] −	2,2’-methylene-bis(6-tert-butyl)-4-ethylphenol	7.255319656	687.7421105	1.47943 × 10^−5^	0.753989673
[M + Hac-H] −	(2-{[3-hydroxy-2-tetradecanamidooctadec-4-en-1-yl phosphonato]oxy}ethyl)trimethylazanium	1.744859227	0.101001266	8.2167 × 10^−5^	1.28624483
[M − H] −	Mestranol	1.55124963	3.498222121	0.000134048	4.974410509
[M − H] −	Rauwolscine	1.590991455	3.222450611	0.000858525	2.253295815
[M − H] −	1-hexadecanoyl-2-(9z-octadecenoyl)-sn-glycero-3-phospho-(1’-myo-inositol)	2.674924291	0.46203907	0.000883256	1.278391498
[M + CH3COOH-H] −	Sm d34:1	3.293558365	0.107293836	0.001682566	0.614405172
[M − H] −	Pi 36:2	2.828081935	0.509520417	0.002043878	0.039202438
[M − H] −	Cis,cis-muconic acid	19.10365688	2.99791356	0.002282177	1.699372541
[M − H] −	2-oleoyl-1-stearoyl-sn-glycero-3-phosphoserine	1.704491502	0.50233312	0.004191965	0.25523065
[M − H] −	Pi(16:0e/15-hete)	1.704070924	0.585164501	0.004289139	1.123702214
[M − H] −	Myo-inositol	1.930136424	0.763514117	0.00430323	0.431600743
[M + Hac-H] −	Pc(16:1e/9-hode)	2.134918492	1.124601576	0.004327026	1.399094575
[M − H] −	2-arachidonoyl-1-palmitoyl-sn-glycero-3-phosphoethanolamine	1.960916534	0.619651975	0.004474611	3.180978267
[M − H] −	3-hydroxy-3-methylglutaric acid	1.910771219	1.879446513	0.007537124	1.331120169
[M − H] −	Glutamic acid	2.692179409	0.642050185	0.008620487	2.04730699
[M − H] −	*N*-acetyl-l-aspartic acid	2.207757039	0.654700304	0.008774719	2.833624788
[M − H] −	Pi 38:4	2.493421846	0.588702225	0.010326359	2.236373454
[M − H] −	Pi 34:2	2.396638201	0.611586826	0.011323699	1.511310532
[M − H] −	3,4-dihydroxyhydrocinnamic acid	2.448942044	2.368625638	0.012071806	3.876790668
[M − H] −	Dl-lactate	2.696831025	0.813506534	0.021585957	5.290769196
[M − H] −	1-stearoyl-2-linoleoyl-sn-glycero-3-phosphoethanolamine	4.046902217	0.535272448	0.027432183	0.476942516
[M − H] −	Pe 32:1	2.224649115	0.60913988	0.031056552	0.638952989
[M − H] −	(2-aminoethoxy)[3-[hexadec-1-en-1-yloxy]-2-[icosa-5.8.11.14-tetraenoyloxy]propoxy]phosphinic acid	5.285454225	0.616442722	0.031251857	0.949210223
[M − H] −	1-palmitoyl-2-oleoyl-phosphatidylglycerol	5.322414542	0.576304139	0.031944236	2.292568905
[M − H] −	Pe(18:1e/12-hete)	2.427631126	0.623348149	0.034032449	5.956456416
[M − H] −	5’-phosphoribosyl-5-amino-4-imidazolecarboxamide (aicar)	1.694809852	1.197983127	0.036763439	4.262055988
[M − H] −	2-linoleoyl-1-palmitoyl-sn-glycero-3-phosphoethanolamine	3.019191108	0.654770708	0.036864178	1.513458381
[M − H] −	Pe(16:1e/15-hete)	2.007563238	0.592051877	0.042176928	2.41362126
[M − H] −	(2-aminoethoxy)[2-[docosa-4.7.10.13.16.19-hexaenoyloxy]-3-[hexadec-1-en-1-yloxy]propoxy]phosphinic acid	4.5379477	0.661536657	0.044480761	0.169100171
[M − H] −	d-mannose	2.054375169	2.637354787	0.048367452	2.341916416

## Data Availability

The data used to support the findings of this study are included within the article.

## Supplementary Materials

The following supporting information can be downloaded at: https://www.mdpi.com/article/10.3390/metabo13010025/s1, Figure S1: Rank value filter. The criterion for determining the best rank value is the leading point of the maximum change in the cophenetic value with K, 3 was choosen. Figure S2: (A) It can be seen that different NMF classes have their own weighted signature. (B) Correlations between 4 genes, Pearson correlation coefficients coded in terms of size and color. (C) The expression of HIF1A, NNMT, OCRL in the test dataset GSE4302. Figure S3: (A) The top 10 enriched GO-BP terms. (B) Differential expression of immune cells in two groups. * *p* < 0.05, ** *p* < 0.01; Table S1: The samples of GSE76262 were respectively clustered in the disease condition and NMF typing. Table S2: Clustering of samples in healthy and diseased groups in GSE4302. Table S3: All DEGs of GSE76262.
