# Supplementary material for: The Combination of Bioinformatics Analysis and Untargeted Metabolomics Reveals Potential Biomarkers and Key Metabolic Pathways in Asthma
Source: Metabolites. 2022 Dec 23;13(1):25. doi: 10.3390/metabo13010025 (PMC9860906; doi:10.3390/metabo13010025)
Supplement: Supplementary file 1 [file metabolites-13-00025-s001.zip › Supplementary table S1--The samples of GSE76262 were respectively clustered in the disease condition and NMF typing.pdf]

Sample	NMF cluster	Disease
GSM1978047	cluster 1	asthma
GSM1978057	cluster 1	asthma
GSM1978098	cluster 1	asthma
GSM1978143	cluster 1	asthma
GSM1978147	cluster 1	asthma
GSM1978152	cluster 1	asthma
GSM1978164	cluster 1	health
GSM1978036	cluster2	asthma
GSM1978037	cluster2	asthma
GSM1978038	cluster2	asthma
GSM1978043	cluster2	asthma
GSM1978045	cluster2	asthma
GSM1978048	cluster2	asthma
GSM1978050	cluster2	asthma
GSM1978051	cluster2	asthma
GSM1978053	cluster2	asthma
GSM1978054	cluster2	asthma
GSM1978055	cluster2	asthma
GSM1978056	cluster2	asthma
GSM1978062	cluster2	asthma
GSM1978064	cluster2	asthma
GSM1978072	cluster2	asthma
GSM1978077	cluster2	asthma
GSM1978085	cluster2	asthma
GSM1978091	cluster2	asthma
GSM1978093	cluster2	asthma
GSM1978094	cluster2	asthma
GSM1978095	cluster2	asthma
GSM1978099	cluster2	asthma
GSM1978101	cluster2	asthma
GSM1978102	cluster2	asthma
GSM1978103	cluster2	asthma
GSM1978104	cluster2	asthma
GSM1978105	cluster2	asthma
GSM1978106	cluster2	asthma
GSM1978107	cluster2	asthma
GSM1978108	cluster2	asthma
GSM1978109	cluster2	asthma
GSM1978110	cluster2	asthma
GSM1978111	cluster2	asthma
GSM1978115	cluster2	asthma
GSM1978119	cluster2	asthma
GSM1978120	cluster2	asthma

GSM1978121	cluster2	asthma
GSM1978123	cluster2	asthma
GSM1978126	cluster2	asthma
GSM1978128	cluster2	asthma
GSM1978129	cluster2	asthma
GSM1978130	cluster2	asthma
GSM1978131	cluster2	asthma
GSM1978133	cluster2	asthma
GSM1978134	cluster2	asthma
GSM1978135	cluster2	asthma
GSM1978136	cluster2	asthma
GSM1978140	cluster2	asthma
GSM1978141	cluster2	asthma
GSM1978142	cluster2	asthma
GSM1978144	cluster2	asthma
GSM1978145	cluster2	asthma
GSM1978146	cluster2	asthma
GSM1978150	cluster2	asthma
GSM1978151	cluster2	asthma
GSM1978153	cluster2	health
GSM1978154	cluster2	health
GSM1978155	cluster2	health
GSM1978156	cluster2	health
GSM1978157	cluster2	health
GSM1978158	cluster2	health
GSM1978159	cluster2	health
GSM1978160	cluster2	health
GSM1978161	cluster2	health
GSM1978162	cluster2	health
GSM1978165	cluster2	health
GSM1978166	cluster2	health
GSM1978168	cluster2	health
GSM1978169	cluster2	health
GSM1978170	cluster2	health
GSM1978171	cluster2	health
GSM1978172	cluster2	health
GSM1978173	cluster2	health
GSM1978035	cluster3	asthma
GSM1978039	cluster3	asthma
GSM1978040	cluster3	asthma
GSM1978041	cluster3	asthma
GSM1978042	cluster3	asthma
GSM1978044	cluster3	asthma
GSM1978046	cluster3	asthma

GSM1978049	cluster3	asthma
GSM1978052	cluster3	asthma
GSM1978058	cluster3	asthma
GSM1978059	cluster3	asthma
GSM1978060	cluster3	asthma
GSM1978061	cluster3	asthma
GSM1978063	cluster3	asthma
GSM1978065	cluster3	asthma
GSM1978066	cluster3	asthma
GSM1978067	cluster3	asthma
GSM1978068	cluster3	asthma
GSM1978069	cluster3	asthma
GSM1978070	cluster3	asthma
GSM1978071	cluster3	asthma
GSM1978073	cluster3	asthma
GSM1978074	cluster3	asthma
GSM1978075	cluster3	asthma
GSM1978076	cluster3	asthma
GSM1978078	cluster3	asthma
GSM1978079	cluster3	asthma
GSM1978080	cluster3	asthma
GSM1978081	cluster3	asthma
GSM1978082	cluster3	asthma
GSM1978083	cluster3	asthma
GSM1978084	cluster3	asthma
GSM1978086	cluster3	asthma
GSM1978087	cluster3	asthma
GSM1978088	cluster3	asthma
GSM1978089	cluster3	asthma
GSM1978090	cluster3	asthma
GSM1978092	cluster3	asthma
GSM1978096	cluster3	asthma
GSM1978097	cluster3	asthma
GSM1978100	cluster3	asthma
GSM1978112	cluster3	asthma
GSM1978113	cluster3	asthma
GSM1978114	cluster3	asthma
GSM1978116	cluster3	asthma
GSM1978117	cluster3	asthma
GSM1978118	cluster3	asthma
GSM1978122	cluster3	asthma
GSM1978124	cluster3	asthma
GSM1978125	cluster3	asthma
GSM1978127	cluster3	asthma

GSM1978132	cluster3	asthma
GSM1978137	cluster3	asthma
GSM1978138	cluster3	asthma
GSM1978139	cluster3	asthma
GSM1978148	cluster3	asthma
GSM1978149	cluster3	asthma
GSM1978163	cluster3	health
GSM1978167	cluster3	health
