# Supplementary material for: The Combination of Bioinformatics Analysis and Untargeted Metabolomics Reveals Potential Biomarkers and Key Metabolic Pathways in Asthma
Source: Metabolites. 2022 Dec 23;13(1):25. doi: 10.3390/metabo13010025 (PMC9860906; doi:10.3390/metabo13010025)
Supplement: Supplementary file 1 [file metabolites-13-00025-s001.zip › Supplementary table S2--Clustering of samples in healthy and diseased groups in GSE4302.pdf]

Sample	Disease
GSM98206	healthy
GSM98207	healthy
GSM98209	healthy
GSM98211	healthy
GSM98212	healthy
GSM98213	healthy
GSM98217	healthy
GSM98218	healthy
GSM98219	healthy
GSM98221	healthy
GSM98222	healthy
GSM98223	healthy
GSM98225	healthy
GSM98229	healthy
GSM98230	healthy
GSM98234	healthy
GSM98235	healthy
GSM98236	healthy
GSM98239	healthy
GSM98240	healthy
GSM98241	healthy
GSM98242	healthy
GSM98243	healthy
GSM98244	healthy
GSM98245	healthy
GSM98251	healthy
GSM98254	healthy
GSM98258	healthy
GSM98141	Asthma
GSM98142	Asthma
GSM98143	Asthma
GSM98144	Asthma
GSM98145	Asthma
GSM98146	Asthma
GSM98147	Asthma
GSM98148	Asthma
GSM98149	Asthma
GSM98150	Asthma
GSM98151	Asthma
GSM98152	Asthma
GSM98153	Asthma
GSM98154	Asthma
GSM98155	Asthma

GSM98156	Asthma
GSM98157	Asthma
GSM98158	Asthma
GSM98159	Asthma
GSM98160	Asthma
GSM98161	Asthma
GSM98162	Asthma
GSM98163	Asthma
GSM98164	Asthma
GSM98165	Asthma
GSM98166	Asthma
GSM98167	Asthma
GSM98168	Asthma
GSM98169	Asthma
GSM98170	Asthma
GSM98171	Asthma
GSM98172	Asthma
GSM98173	Asthma
GSM98174	Asthma
GSM98175	Asthma
GSM98176	Asthma
GSM98177	Asthma
GSM98178	Asthma
GSM98179	Asthma
GSM98180	Asthma
GSM98181	Asthma
GSM98182	Asthma
GSM98183	Asthma
GSM98184	Asthma
GSM98185	Asthma
GSM98186	Asthma
GSM98187	Asthma
GSM98188	Asthma
GSM98189	Asthma
GSM98190	Asthma
GSM98191	Asthma
GSM98192	Asthma
GSM98193	Asthma
GSM98194	Asthma
GSM98195	Asthma
GSM98196	Asthma
GSM98197	Asthma
GSM98198	Asthma
GSM98199	Asthma

GSM98200	Asthma
GSM98201	Asthma
GSM98202	Asthma
GSM98203	Asthma
GSM98204	Asthma
GSM98208	Asthma
GSM98210	Asthma
GSM98214	Asthma
GSM98215	Asthma
GSM98216	Asthma
GSM98220	Asthma
GSM98224	Asthma
GSM98226	Asthma
GSM98227	Asthma
GSM98228	Asthma
