# Supplementary figures and images for: The Combination of Bioinformatics Analysis and Untargeted Metabolomics Reveals Potential Biomarkers and Key Metabolic Pathways in Asthma
Source: Metabolites. 2022 Dec 23;13(1):25. doi: 10.3390/metabo13010025 (PMC9860906; doi:10.3390/metabo13010025)

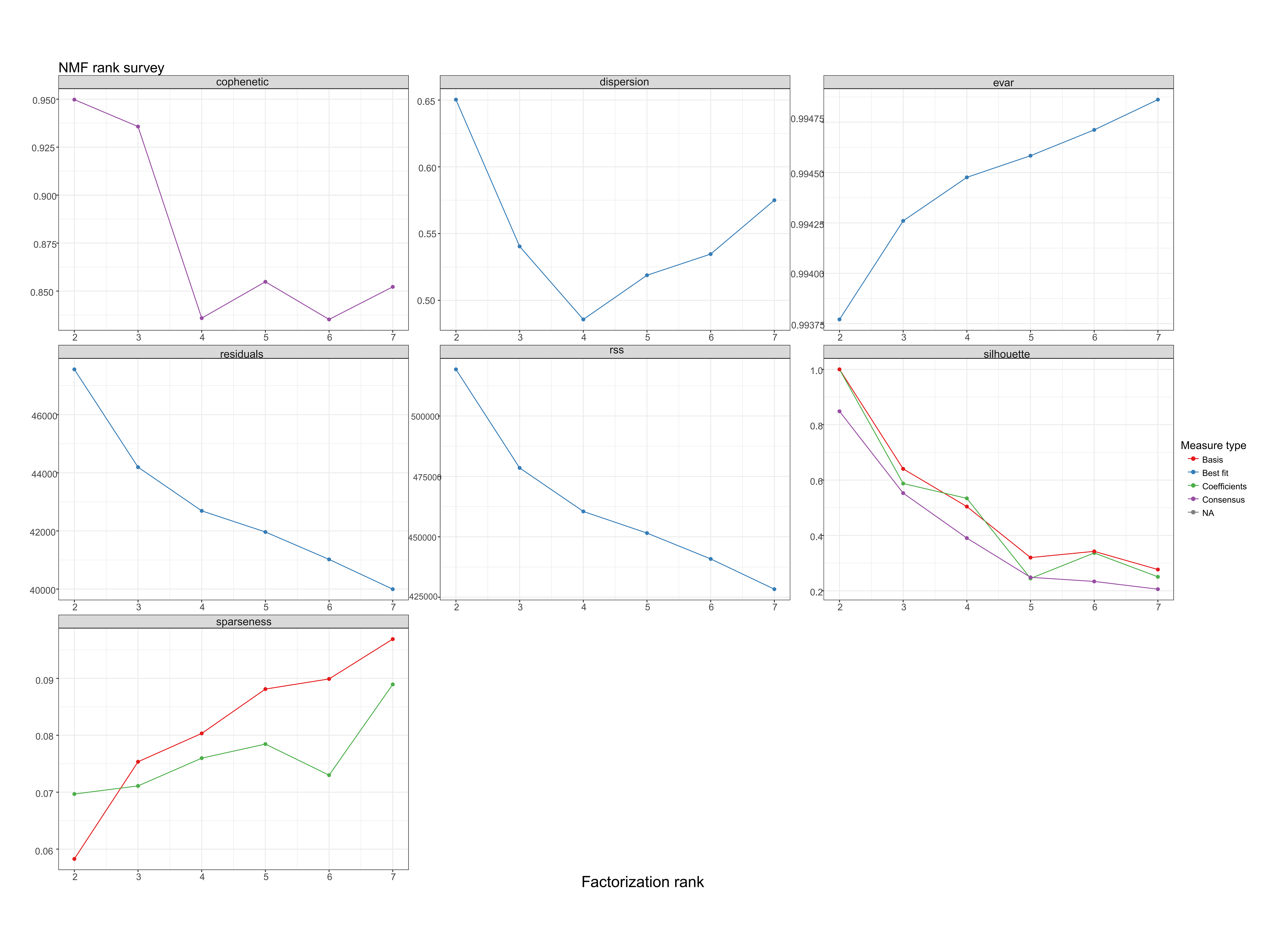

Supplement: Supplementary file 1 [file metabolites-13-00025-s001.zip › Supplementary figure S1.tif]

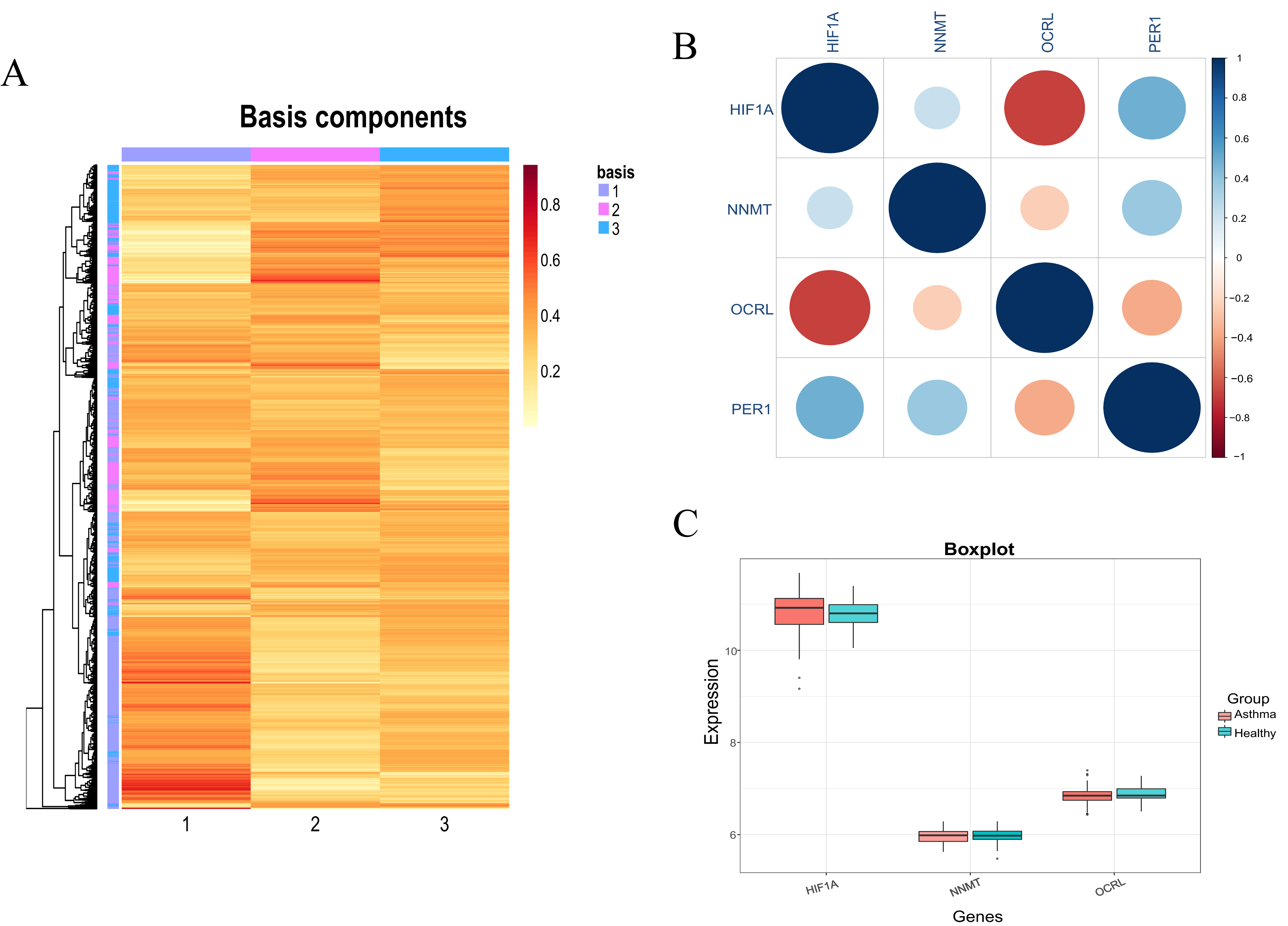

Supplement: Supplementary file 1 [file metabolites-13-00025-s001.zip › Supplementary figure S2.tif]

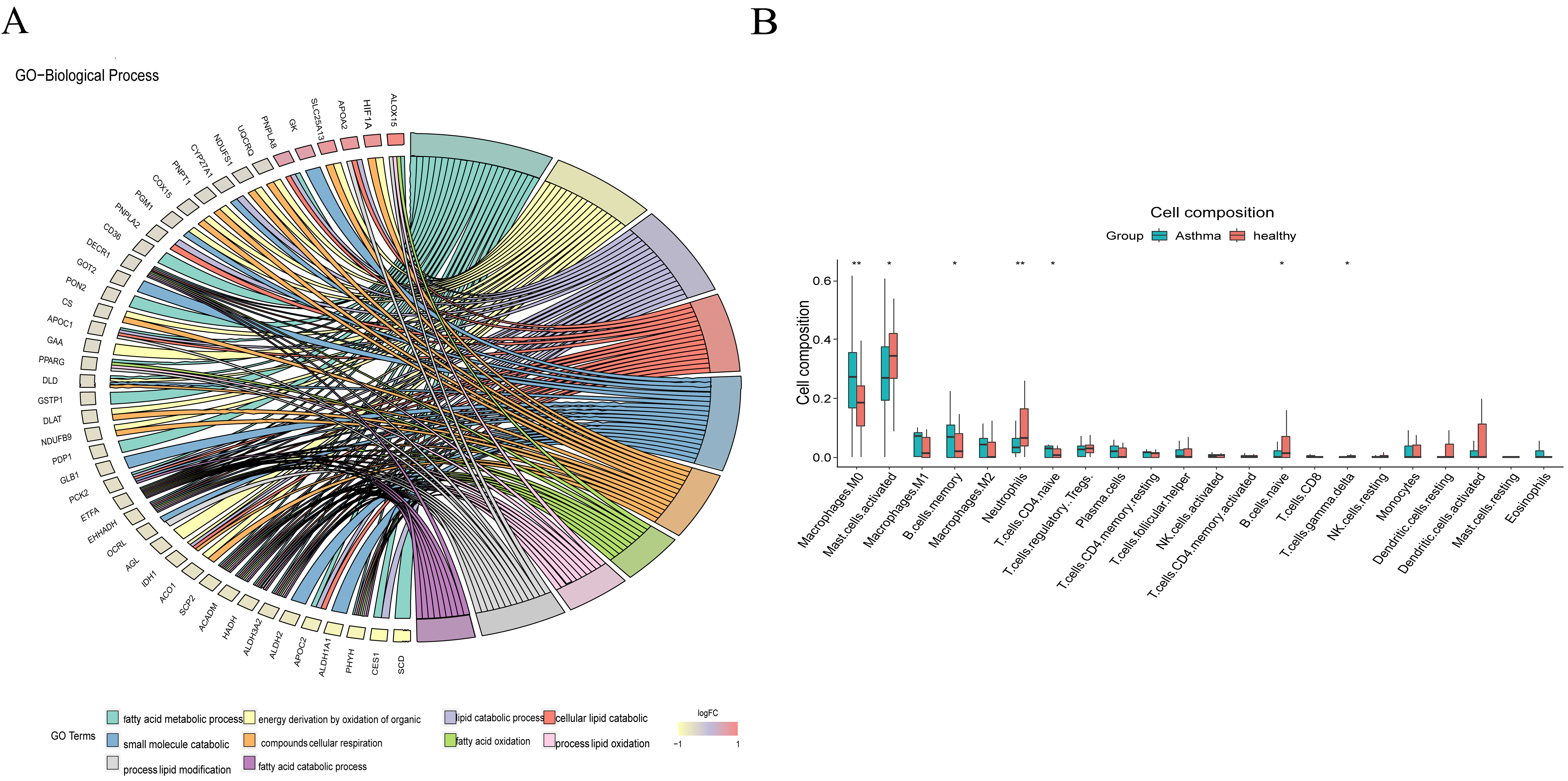

Supplement: Supplementary file 1 [file metabolites-13-00025-s001.zip › Supplementary figure S3.tif]
